# Death Penalty and Psychiatric Evaluation in Japan

**DOI:** 10.3389/fpsyt.2018.00550

**Published:** 2018-11-02

**Authors:** Hiroko Kashiwagi, Naotsugu Hirabayashi

**Affiliations:** Department of Forensic Psychiatry, National Center Hospital of Neurology and Psychiatry, Kodaira, Japan

**Keywords:** death penalty, psychiatric evaluation, testimony, lay judge system, delusion, MTSA

## Abstract

Japan recently ordered a string of death sentences for offenders with mental illness. Based on the verdicts, we describe cases where one or more psychiatrists conducted psychiatric evaluations for several months and testified in lay judge courts. We compared these cases with those in which the death penalty was avoided, or the mandating treatment order was applied. Additionally, we discuss a trend toward more severe punishment and Japanese cultural background seen in a public opinion survey. Moreover, we introduce a research report that concluded a strong correlation between the number of victims and death penalty verdict. In Japan, lay judge trials determine the sentencing of the defendant and the verdict of guilty or not guilty, and it can be difficult for psychiatrists to help lay judges understand psychiatric symptoms and the relationship between symptoms and criminal responsibility through their testimony. We believe the right to life is the most fundamental of human rights and that the death penalty is inhumane. The death penalty also eliminates the possibility of treatment or rehabilitation, despite the fact that psychiatrists should support the possibility of treatment or rehabilitation in all cases. Further, the Japanese Penal Code does not permit execution for those mentally ill deemed unable to receive sentence; however, it is unclear who will conduct these evaluations and how they will do so. We describe our beliefs of how psychiatrists should act in these situations.

## Introduction

In 2009, the International Federation for Human Rights and the Center for Prisoners' Rights wrote to the Japanese government, expressing strong concerns about executions in Japan, particularly of people with mental illness (1). Almost 10 years later, Japan ordered a series of death sentences for offenders with mental illness. Despite a global trend toward abolition of the death penalty, Japan is one of a minority of countries that continue to resort to executions (2). Moreover, a trend toward more severe punishment has been indicated in Japan. After the 2008 introduction of the participating victim system and 2009 introduction of lay judge trials, there has been some difficulty in the testimony of a psychiatrist who conducted a psychiatric evaluation to assist lay judges in understanding the defendant's psychopathology and determine a professional sentence. We describe cases, raise problems in practices and ethical points of view, and provide future recommendations.

## Methods

We present the cases below mainly based on verdicts of courts compiled by the TKC Law Library online service. The TKC Law Library compiles verdicts into a database and provides them for academic use with a fee. Although verdicts are not subject to copyright according to Article 13 of the Copyright Act, we obtained written approval to quote the verdicts from the company. Additionally, in quoting verdicts, we include the LEX/DB reference number and the name of the court and verdict date. We also discuss open access webpages, articles, and a book review.

## Recent cases involving the death penalty

In May 2015, a man who killed seven people was sentenced to death by the Supreme Court in Japan (3). The man thought he was being monitored and that his neighbors were speaking badly about him behind his back. He was diagnosed with delusional disorder, and in his psychiatric evaluation his persecutory delusions were considered to influence the crime. However, the verdict concluded that his motive for the crime was understandable in terms of his personality and the fact that he had had conflict with the neighbors since childhood, and that the influence of his delusional disorder on the crime was small, resulting in the decision that he had full responsibility [Osaka High Court (April 26, 2015) LEX/DB25540670]. In September 2016, a man who murdered five neighbors was sentenced to death in his second trial. He thought rumors were being spread about him and felt provoked by his neighbors. Once, he believed his curry was poisoned with pesticides. The verdict was that despite being diagnosed with delusional disorder in a psychiatric evaluation, his delusions did not make him feel as though his life was threatened during the crime, and his values and personality had more influence on the degree of the retaliation than his delusions, resulting in a decision that he had full responsibility [Hiroshima High Court (September 13, 2017) LEX/DB25543809]. In March 2017, a man diagnosed with methylphenidate-induced psychosis in psychiatric evaluation (with delusions that continued several years after stopping use, in which case the diagnosis could be changed) was sentenced to death in his initial trial (lay judge trial). After his search for the cause of his hallucinations of bodily sensation, delusional intuition, and delusional percept, he believed he was being attacked by the Japanese government and covert operatives in an act of “psychological technological war,” and killed five neighbors whom he believed to be the covert agents (4). The victims' bereaved families responded saying “nothing but the death sentence is acceptable.” The defendant, however, denied the allegations during the trial, saying he was “manipulated by agents with magnetic waves” (5). The verdict stated that his delusions did not make him feel that his life was threatened during the crime, and his delusions and hallucinations did not promote his crime directly, because his motive for the crime was to reveal the existence of “psychological technological war,” which meant he had other choices. The verdict also stated that he had some degree of planning ability during the crime; his world view and distorted sense of justice influenced the crime; and his psychosis did not influence the murder itself, based on the psychiatric evaluation, resulting in a decision that he had full responsibility [Kobe District Court (March 22, 2017) LEX/DB25448600]. In March 2018, a Peruvian man who killed six people received the death sentence after a two-day jury deliberation of testimony by a psychiatrist who evaluated and diagnosed him with schizophrenia. The man believed he was being chased by men in black suits who would kill him and his relatives; so he escaped from them, lost his money, committed a robbery and murder, and stole money and a car. He also committed a sex crime and robbery in another home; after the arrival of a police officer, he cut his arm with a kitchen knife and jumped from a window. In the lay judge trial, the victims' bereaved families demanded the death penalty for the defendant, who continually showed incoherent speech in court. The verdict stated that he had full responsibility for the crime because his motive for the murder was robbery and he did not kill the man whom he believed to be following him; therefore, his schizophrenia did not overwhelmingly influence the crime. Additionally, it was judged that he recognized the illegality of the crime due to evidence of his concealment of the crime [Saitama District Court (March 9, 2018) LEX/DB25560015]. In March 2018, a caregiver who killed three people in an elderly nursing home by pushing them off a balcony was sentenced to death in his initial trial. On psychiatric evaluation, he was diagnosed with autism spectrum disorder and intellectual deficits (IQ = 68). He was subject to violent language from his first victim, who he killed. After the crime, he felt satisfied that his cardiopulmonary resuscitation (CPR) performance was viewed by other people, and planned to kill two more people to display his CPR performance to other people. He changed testimony repeatedly in the judicial proceedings, and finally denied his crime in lay judge court. The bereaved families criticized the defendant's “lack of remorse” and demanded the death penalty. The verdict stated that his crime was partly explained by mental illness, yet could also be explained by the portion of his mind that was not mentally ill, thus resulting in a decision that he had full responsibility. As he had no remorse, there was no possibility of rehabilitation [Yokohama District Court (March 22, 2018) LEX/DB25560322].

In all cases, psychiatric evaluations were performed by one or more psychiatrists for several months, and psychiatrists' testimony was taken in lay judge courts. Although diagnoses of the various mental disorders noted above were recognized, the defendants were judged to have full criminal responsibility and were sentenced to death without any reduction in punishment in light of extenuating circumstances. In the former four cases, it is questionable whether the defendants recognized the meaning, nature, and anti-morality of their actions due to delusions, despite that point influencing their criminal responsibility directly. It is likely that lay judges were not able to comprehend how ill these offenders were. In the last case, lack of remorse was recognized as a predictor of the impossibility of rehabilitation; we feel this is unacceptable from a professional point of view, because many predictors other than remorse indicate the possibility of rehabilitation. In Japan, Article 39 of the Penal Code stipulates that insanity precludes guilt, positioning diminished responsibility as a reason for the reduction in punishment. Additionally, mental illness, as a general condition, could serve as an extenuating circumstance for a reduced sentence (Article 66 of the Penal Code). However, it is important that the possibility of treatment and rehabilitation of the defendant was not stated in each verdict stated above.

## Mass murder cases avoiding the death penalty

### Cases from 1983 to 1984

In trials from 1983 to 1984, even in cases of mass murder, diminished responsibility was used as grounds for avoiding the death penalty (6). In November 1983, a man diagnosed with schizophrenia who killed five people, was sentenced to life imprisonment due to diminished responsibility. He killed a woman (and her family) who refused his marriage proposal due to differences in ideology. The verdict stated that his schizophrenia was well controlled with medication. Furthermore, his planning ability during the crime and concealment after the crime were recognized; nevertheless, schizophrenia symptoms were believed to influence his motive and manner of the crime [Takamatsu High Court (November 2, 1983) LEX/DB24005982, Kochi District Court (24 April 1970) LEX/DB24005513]. In April 1984, a man who killed six people was sentenced to life imprisonment due to diminished responsibility. He thought he was being tricked by passersby and set fire to a bus. He was diagnosed with mild intellectual disability (IQ = 69) on psychiatric evaluation, and it was believed that drinking might have also influenced the crime (Tokyo District Court (April 24, 1984) LEX/DB27917096). In December 1984, a man killed six people and was sentenced to life imprisonment due to diminished responsibility. He thought his neighbors' despising him had created his unsociable personality, and shot the neighbors to death with a hunting gun. In several psychiatric evaluations, he was diagnosed with schizophrenia and delusional disorder. The verdict stated that despite his planning the crime, persecutory delusions influenced the crime and he had diminished responsibility [Takamatsu High Court (December 4, 1984) LEX/DB27921929]. In each case, the death penalty was demanded by prosecutors but was avoided after recognizing diminished responsibility. In these cases, the existence of schizophrenia or delusions was highlighted, the manner and degree of psychiatric symptoms' influence on the crime was analyzed in less detail, and the defendant's capacities and lack of mental illness were less likely to be highlighted compared to the recent verdicts summarized in the previous section.

### Cases following the MTSA

In Japan, the Medical Treatment and Supervision Act (MTSA) mandated the provision of professional treatment to mentally disordered offenders and has been in force since 2005. As of 2018, 33 hospital wards and 833 beds have been made available in designated inpatient facilities across Japan, with a further 601 hospitals (including clinics) maintaining designated outpatient facilities (7). The MTSA applies to individuals who have committed serious offenses such as homicide, bodily injury, arson, robbery, rape, and forcible indecency and are determined to have been insane or have had diminished responsibility due to a mental disorder during the crime. The prosecution can file an MTSA motion against mentally disordered offenders exempted from prosecution, for whom indictment is suspended, or who are judged innocent or given a suspended sentence, enabling them to receive involuntary psychiatric treatment. According to statistics in the White Paper on Crime issued by the Ministry of Justice 2016, prosecutors filed MTSA motions in 350 cases (of which 313 were exempted from prosecution, three yielded innocent verdicts, and 34 resulted in suspended sentences). Among these, the decision to hospitalize under the MTSA was taken in 238 cases, while outpatient treatment was mandated in 36 cases (8). Thus, most cases treated under the MTSA are those in which charges are dropped following psychiatric evaluation at the prosecution stage. This is associated with the fact that more than 99.9% of cases in Japanese courts pursued by the prosecution result in convictions (9). The total number of individuals arrested for homicide in 2016 was 816, 121 of whom had (or were suspected to have) mental illness (10). Of these, the prosecution filed motions under the MTSA in 96 cases (87 exempted from prosecution, one innocent, eight suspended sentences), with the decision to hospitalize taken in 69 cases and outpatient treatment mandated in 10 cases (8).

Under this act, offenders who previously suffered from mental disorders have received treatment and been reintegrated into society. Additionally, it is notable that among cases of individuals hospitalized at the MTSA-designated ward (66 beds) with which the authors are affiliated—the first such ward to be opened in Japan 13 years ago–there have been five cases of multiple homicide (two–three victims). In four such cases, the victims were family members, while the remaining case involved an individual who set fire to a group home, with its residents being the victims.

### A case in which the death penalty was avoided while acknowledging full criminal responsibility

Moreover, among cases for which the prosecutors sought the death penalty, there were instances where, while acknowledging the defendant's full criminal responsibility, sentencing decisions nonetheless considered the influence of mental illness. In an incident involving the murder of two men in Osaka in May 2010, the Osaka District Court, while acknowledging that “schizophrenia was being well managed with treatment,” did not grant the death penalty partly because delusions caused by schizophrenia were linked to the motivation for the murders, resulting in a life sentence at the first trial in December 2012. In March 2014, the Osaka High Court dismissed an appeal, affirming the sentence.

## Discussion

### Correlation between number of victims and death penalty verdict

According to a research report by the Supreme Court Legal Training and Research Institute, among the 346 cases for which the death penalty was sought and that resulted in a sentence of either the death penalty or life imprisonment between 1970 and 2009 (193 death penalties, 153 life imprisonment cases), the proportion for which the death penalty was affirmed was 32% in cases involving only one victim, 59% in cases involving two victims, and 79% in cases involving three or more victims (6). The researchers concluded that “there is a strong correlation between the number of victims and death penalty verdict” (6).

### Changes in policy and the penal code

Underlying the continued delivery of death penalty verdicts for persons with mental disorders was the 2008 introduction of the participating victim system, lay judge trials in 2009, normalization of persons with mental disorders, and normalization of criminal responsibility.

### Trend toward severe punishment and japanese cultural background

A trend toward more severe punishment has also been indicated. According to a public opinion survey, the proportion of citizens agreeing that “the death penalty should be abolished in all cases” peaked at 20.7% in 1975 and has since been decreasing gradually (6). One turning point in this trend toward more severe punishment is said to be a series of incidents involving the apocalyptic cult Aum Shinrikyo in 1989 to 1995 (6). In terms of characteristics specific to Japan, cultural background has also been considered important in terms of emphasis on the presence or absence of remorse and the bereaved family's feelings (11). In a Cabinet Office poll in 2014, 80.3% respondents accepted that “the death penalty is unavoidable” while 9.7% rejected this, affirming that “the death penalty should be abolished” (12). The most frequent reason given for accepting the death penalty (from multiple-choice responses) was that “the feelings of the victims and their families cannot be contained” (53.4%), followed by “atrocious crimes should be paid for with life” (52.9%), and “there is a danger that perpetrators might commit similar crimes if allowed to live” (47.4%) (12).

### Difficulties in the testimony of a psychiatrist in a lay judge trial

In Japan, lay judge trials determine the sentencing of the defendant. Therefore, there is some difficulty in the testimony of a psychiatrist who conducted a psychiatric evaluation to assist the lay judges in understanding the defendant's psychopathology and determine a professional sentence. Criminal responsibility is a judicial determination, which courts are given the freedom to make without being restricted by psychiatrists' reports. Cases with a large social impact, including those with many victims or where the victims are people other than family members, are less likely to have reduced sentences even if mental illness has a bearing on the crime. In cases of the killing of multiple others, even if the motivation is influenced by psychiatric symptoms including delusions, multiple murders could be accomplished by retaining some degree of executive function, and in many cases, planning will be admitted. Even in such cases, while the death penalty would have been avoided in the 1980s, the situation has changed recently. In recent verdicts, the manner and degree of psychiatric symptoms' influence on the crime are analyzed in more detail, and the defendant's capacities and lack of mental illness are likely to be highlighted. It is difficult and complicated for lay judges to understand both, the psychosis and the complicated relationship between psychosis and the crime, and make decisions.

The influence of mental disorders may make it difficult for individuals to express apologies or remorse in court; for example, remorse can be difficult due to delusions or disturbances of thought. Furthermore, these individuals may be pathologically unable to imagine others' feelings or circumstances. Beyond the perception of there being no possibility of rehabilitation, they may further hurt the feelings of the bereaved, which may, in turn, affect the lay judge's decision. Additionally, individuals with autistic spectrum disorder or intellectual disorder are likely to fail in defense in each stage of the judicial proceedings including testimony in court.

### Ethical issues related to the death penalty

Some ethical issues exist regarding the death penalty for offenders with mental illness (13, 14). The World Medical Association (WMA) and World Psychiatric Association (WPA) stated that it is unethical for physicians to participate in capital punishment (15, 16). In Japan, Article 479 of the Penal Code forbids the execution of inmates with insanity who are found unable to receive sentence on account of mental illness. However, it is unclear who will evaluate and how they will do so to determine this. A potential dilemma is whether inmates with mental illness should receive treatment, as both outcomes are contrary to medical ethics. In 2002, The Japanese Society of Psychiatry and Neurology opined that psychiatrists should not be involved in treatment or psychiatric evaluations that may result in execution because a death row inmate who is no longer deemed insane may then receive sentence and be executed. In 2004, the society stated that its attitude toward the maintenance or abolition of capital punishment was pending, and that it would try to grasp the actual circumstances of persons sentenced to death or death row. However, almost 15 years later, Japan has continued with executions in secret, and the situation has not changed.

Japan executes by hanging; prisoners are not informed of their execution date. We believe the right to life is the most fundamental of human rights and that the death penalty is inhumane. The death penalty eliminates the possibility of treatment or rehabilitation, despite the fact that psychiatrists should support the possibility of treatment or rehabilitation in all cases.

## Recommendations

-Psychiatrists should create an easy-to-understand presentation for lay judges to enable them to understand how ill these offenders are.

-Psychiatrists should explain the psychopathology of the defendant's behavior and speech in courts, in instances where they appear to have no remorse or could hurt the feelings of the bereaved.

-In recent verdicts related to the death penalty, the necessity and possibility of treatment is neither highlighted nor mentioned. Ethically, treatment and penalties should be provided when necessary. Furthermore, psychiatrists should highlight the necessity and possibility of treatment of defendants in psychiatric evaluations and testimony, and demand that these points be reflected in the verdict and with respect to criminal responsibility.

-Moreover, decisions on the treatment possibility likely depend on recent developments in psychiatric treatment. Our opinion is that psychiatrists should be keen to develop effective treatments for mentally disordered offenders such as those who commit mass murder due to mental illness, with personality or developmental disorders, and for whom the MTSA applies (more than 80% of individuals are diagnosed with schizophrenia); then, they should provide results, and appeal to public opinion. Furthermore, psychiatrists must be conscious of potential effects of changes in diagnostic criteria and treatments on the death sentence.

-We would like to demand the disclosure of information regarding the situation of death row or persons with mental illness sentenced to the death penalty.

-A parallel development would entail psychiatric collaboration with organizations supporting the bereaved family or victims.

## Conclusion

We presented cases both involving the death penalty and those in which the death penalty was avoided. We found cases with a large social impact, including those with many victims or where the victims were people other than family members, were less likely to have reduced sentences even if mental illness had a bearing on the crime. From an ethical point of view, the death penalty is never acceptable for us as both physicians and psychiatrists. Interdisciplinary discussions are needed from scientific, medical, legal, political, and ethical perspectives before allowing continuation of death sentences for offenders with mental illness.

## Author contributions

HK contributed to drafting the manuscript. NH contributed to the conception of the manuscript. All authors approved the final version of the manuscript.

### Conflict of interest statement

The authors declare that the research was conducted in the absence of any commercial or financial relationships that could be construed as a potential conflict of interest.
